# Combined on- and off-site training contributes to strengthening the unified pharmaceutical system in the Dominican Republic

**DOI:** 10.1186/2052-3211-7-S1-O24

**Published:** 2014-12-17

**Authors:** Edgar Barillas, Claudia Valdez, Paula Diaz, Maria Elena Tapia

**Affiliations:** 1 Systems for Improved Access to Pharmaceuticals and Services (SIAPS), Management Sciences for Health (MSH), Santo Domingo, Dominican Republic; 2 National Pharmaceutical System, Ministry of Health, Santo Domingo, Dominican Republic

## Background

With support from USAID-funded projects, the Dominican Republic (DR) started organizing a unified pharmaceutical system (SUGEMI) in 2010. Implementing SUGEMI included developing standard operating procedures (SOPs) for all system components, appointing personnel for national and regional pharmaceutical units, and training on-site staff responsible for pharmaceutical supply management. Strengthening and sustaining SUGEMI is now dependent on in-depth training of public health system staff responsible for supply management functions.

## Method

SIAPS involved key personnel early in the elaboration of SOPs and training activities and helped design and implement a 12-week on-site/off-site certificate course on pharmaceutical supply. Each of the six program modules includes preparatory activities (reading Management Sciences for Health’s Managing Drug Supply textbook and SUGEMI SOPs); on-site sessions (discussing readings and instructions for on-the-job practice), and on-the-job site practice (situation analysis of the students’ institutions and identification of alternative interventions to address problems).

## Results

Rapid capacity building has contributed to a nationwide implementation of SUGEMI in less than three years. Major outcomes were: National and Regional Pharmaceutical Unit staff members were trained to replicate trainings for SUGEMI implementation in 1,105 primary care facilities and 143 hospitals, and two on-site/off-site site courses have been completed (2012/2013 public university course for 35 students, and 2013/2014 private university course for 33 students). All students successfully fulfilled the academic requirements to obtain their certificates. Half of the graduates in the first course and all in the second were employed in a public health facility.

## Discussion

Basic training in operational procedures is a necessary first step when implementing a national pharmaceutical system. However, consolidation and sustainability demands professionals with in-depth knowledge of concepts and tools commonly used in supply management. A hybrid on-site/off-site approach directed toward health workers in the public sector assures: reinforcement of the theory through practical experience, implementation of a national pharmaceutical system, and immediate introduction of good pharmaceutical management practices in their particular labour sites. All trainings had an immediate operative purpose–the implementation of particular SUGEMI component—fixing knowledge, through practice.

## Lessons learned

Implementing a national pharmaceutical system offers a unique opportunity to consolidate theoretical concepts with practical on-the-job experiences. Involving personnel in the elaboration and training in implementing SOPs, and an on-site/off-site course simultaneously strengthens SUGEMI and builds capacity of personnel in pharmaceutical supply management.

